# Berotralstat and Health‐Related Quality of Life in Hereditary Angioedema: Pooled Analysis of the APeX‐2 and APeX‐J Trials

**DOI:** 10.1111/cea.70274

**Published:** 2026-04-26

**Authors:** Teresa Caballero, Sorena Kiani‐Alikhan, Douglas T. Johnston, Aolin Wang, Lorena Lopez‐Gonzalez, Zheng‐Yi Zhou, Fan Yang, Hannah H. Kim, Sandra Nestler‐Parr, Dianne Tomita, Patrick Gillard, Tamar Kinaciyan

**Affiliations:** ^1^ Allergy Department Hospital Universitario La Paz, IdiPAZ, CIBERER U754 Madrid Spain; ^2^ Department of Immunology Royal Free London NHS Foundation Trust London UK; ^3^ BioCryst Pharmaceuticals, Inc. Durham North Carolina USA; ^4^ Analysis Group, Inc. New York New York USA; ^5^ Analysis Group, Inc. London UK; ^6^ Department of Dermatology Medical University of Vienna Vienna Austria

**Keywords:** AE‐QoL, APeX trials, berotralstat, health‐related quality of life, hereditary angioedema, patient‐reported outcome measures

## Abstract

**Background:**

In the phase 3 APeX‐2 and APeX‐J trials, improvements in health‐related quality of life (HRQoL) were reported for berotralstat, the only oral, once‐daily, long‐term prophylaxis for hereditary angioedema (HAE). We conducted a post hoc trial analysis to evaluate the impact of berotralstat on HRQoL versus placebo through 24 weeks, and the long‐term effects of treatment up to 96 weeks, using pooled APeX‐2 and APeX‐J patient data.

**Methods:**

Patients receiving berotralstat 150 mg or placebo using the Angioedema QoL (AE‐QoL) questionnaire; total and domain scores were analysed. Changes from baseline through Week 24 were compared using mixed models for repeated measures (MMRM) to estimate least squares mean differences (LSMDs) with 95% confidence intervals (CI) and nominal *p*‐values. After Week 24, in the absence of a placebo comparator, long‐term changes from baseline were evaluated using within‐subject effect sizes (Cohen's *d*) through Week 96 in patients receiving berotralstat. Clinical meaningful improvements in AE‐QoL total and domain scores were assessed by minimal clinically important difference (6‐point reduction in AE‐QoL total score) and distributional criterion methods, respectively. The manufacturer of berotralstat (BioCryst Pharmaceuticals) funded this study.

**Results:**

Overall, 47 and 45 patients received berotralstat and placebo, respectively. At Week 24, berotralstat showed significantly greater improvement in AE‐QoL total (LSMD: −7.4; 95% CI: −14.3, −0.6; *p* < 0.05), functioning domain (LSMD: −10.9; 95% CI: −19.6, −2.3; *p* = 0.013) and fears/shame domain (LSMD: −8.7; 95% CI: −17.2, −0.2; *p* = 0.046) scores compared with placebo. Among patients receiving berotralstat, consistent sustained improvements were observed across all AE‐QoL domains up to Week 96. Analyses of meaningful improvement in total score and domains showed an increase over time in the proportion of patients achieving meaningful change.

**Conclusion:**

Berotralstat significantly improved HRQoL versus placebo through 24 weeks and showed long‐term beneficial effects through 96 weeks.

## Key Messages

- At Week 24, berotralstat demonstrated significantly greater improvement in AE‐QoL total score compared with placebo.
- Berotralstat significantly improved functioning and fears/shame AE‐QoL domain scores versus placebo at Week 24.
- Improvements with berotralstat in AE‐QoL total and domain scores were sustained through Week 96.

## Graphical abstract

Pooled analysis of the phase 3 APeX‐2 and APeX‐J trials evaluated the impact of berotralstat 150 mg on health‐related quality of life in patients with hereditary angioedema using the Angioedema Quality of Life (AE‐QoL) questionnaire. Berotralstat significantly improved AE‐QoL total and domain scores for daily functioning and fears/shame versus placebo through Week 24. Improvements in the berotralstat group were sustained through Week 96.

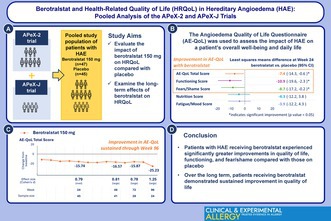

## 1 Introduction

Hereditary angioedema (HAE) is a genetic disorder characterised by recurrent episodes of skin, gastrointestinal tract, and upper airways edema. It is predominantly caused by SERPING1‐mutation‐induced C1‐inhibitor (C1‐INH) dysfunction, although forms with normal C1‐INH and other gene mutations also exist. In all types, symptoms are primarily mediated by bradykinin overproduction [1, 2]. Although HAE is rare, with an estimated global prevalence of 0.13–1.6 cases per 100,000, it imposes a substantial burden, often worsened by misdiagnosis and delayed recognition owing to its nonspecific symptoms and low awareness among healthcare professionals [3].

Swelling episodes from bradykinin excess can be painful, disfiguring, debilitating, and potentially life‐threatening, with considerable impact on patients' daily functioning and health‐related quality of life (HRQoL) [3, 4]. The unpredictability of the swelling episodes further contributes to substantial emotional and psychological burden, including increased anxiety, fear, and social limitations [4]. There remains a critical unmet need for long‐term prophylactic strategies that minimise the impact of HAE on patients' lives.

Berotralstat, a highly selective plasma kallikrein inhibitor, is the first and only oral once daily (QD) therapy approved in the United States (US), European Union, and Japan for the prophylactic treatment of swelling attacks in adults and paediatric patients aged ≥ 12 years with HAE [5, 6]. Approval in the US and Europe was based on the results from the pivotal phase 3 APeX‐2 trial (NCT03485911) in which berotralstat 150 mg achieved clinically meaningful reductions in HAE attack frequency at 6 months, with sustained efficacy through 12 months; mean monthly attack rate decreased from 2.9 at baseline to 1.0 [7]. Similar results were observed in the phase 2 open‐label APeX‐S study (NCT03472040), with a mean monthly attack rate of 0.8 after 12 months [8]. Recent findings from the long‐term phase 3 APeX‐J (NCT03873116) trial conducted in Japan demonstrated an 84.8% mean reduction in HAE episodes from baseline to 26 months [9]. Across studies, berotralstat had an acceptable safety profile and was generally well tolerated [7, 8, 9, 10].

HRQoL also improved with berotralstat. In both APeX‐2 and APeX‐J, 43% of patients receiving berotralstat 150 mg achieved minimal clinically important difference (MCID) in Angioedema QoL (AE‐QoL) total score, compared with 17% in the placebo group at 6 months (least square mean difference [LSMD] of change from baseline: −4.9 in APeX‐2 [*p* = 0.188]; −19.0 in APeX‐J [nominal *p* = 0.061]) [7, 11]. In APeX‐J, clinically meaningful improvements in AE‐QoL total and domain scores from baseline were maintained through 26 months, and treatment satisfaction increased with berotralstat prophylaxis over this period [9]. While these studies were not specifically powered to detect statistically significant differences in HRQoL, both suggest a HRQoL benefit associated with berotralstat treatment in patients with HAE. Building on these observations, this study pooled APeX‐2 and APeX‐J data to evaluate the impact of berotralstat 150 mg on HRQoL compared with placebo through 24 weeks and the long‐term effects of treatment up to 96 weeks.

## 2 Methods

### 2.1 Data Source

The design and methodology of the APeX‐2 and APeX‐J trials are described in detail elsewhere [7, 10, 11]. Both were phase 3, randomised, double‐blind, placebo‐controlled, parallel‐group studies evaluating the efficacy and safety of berotralstat for prevention of HAE attacks using comparable inclusion and exclusion criteria. APeX‐2 enrolled patients from the US, Canada, and several European countries, while APeX‐J enrolled patients from Japan [7, 11].

Both trials randomised participants 1:1:1 to receive QD berotralstat 150 mg, berotralstat 110 mg or placebo, and followed a three‐part structure. In Part 1 (Week 1–24), patients received their initially assigned treatment. At the end of Week 24, patients in the placebo group who completed Part 1 entered Part 2, where they were re‐randomised 1:1 to receive either berotralstat 150 mg or berotralstat 110 mg; Part 2 continued through Week 48 in APeX‐2 and Week 52 in APeX‐J. In Part 3, all patients received open‐label berotralstat 150 mg. The open‐label extension continued through Week 144 in APeX‐2 and Week 104 in APeX‐J [7, 11].

This pooled analysis was restricted to patients receiving either berotralstat 150 mg or placebo during Part 1, prior to treatment reassignment. The long‐term HRQoL trajectory was evaluated among patients receiving berotralstat 150 mg who were followed for up to 96 weeks, based on the standardised HRQoL assessment schedule.

The manufacturer of berotralstat (BioCryst Pharmaceuticals) funded the study.

### 2.2 Study Population

Although APeX‐2 and APeX‐J differed in geographic location, both used similar eligibility criteria, enabling the pooling of corresponding treatment arms for this analysis. Males and non‐pregnant, non‐lactating females aged ≥ 12 years with confirmed diagnosis of Type I or II HAE and access to standard of care (SOC) were included. Patients were also required to have experienced ≥ 2 distinct HAE attacks during a 56‐day run‐in period prior to randomization. Each attack was confirmed by the investigator or expert adjudicator per study protocol and had been treated, required medical attention, or resulted in functional impairment as recorded by the patient. Patients were excluded if they had received prophylactic androgens or tranexamic acid within 28 days prior to screening, or prophylactic exogenous C1 inhibitors within 14 days prior to screening.

The analytical sample comprised the safety populations from APeX‐2 and APeX‐J, including all patients who were randomised to and received ≥ 1 dose of berotralstat 150 mg (berotralstat cohort) or placebo (placebo cohort). Patients in the safety set differed from the intent‐to‐treat population by one patient who was randomised to placebo but did not receive any study drug [10, 11]. All patients or their caregivers in APeX‐2 and APeX‐J trials provided written informed consent or assent before study initiation.

### 2.3 Outcomes

Change in AE‐QoL scores from baseline was assessed at scheduled follow‐up visits at Weeks 4, 8, 12, 18, 24, 28, 32, 36, 48, 60, 72, 84, and 96 in both APeX‐2 and APeX‐J. The AE‐QoL is a validated, angioedema‐specific tool comprising 17 items that measure HRQoL across four domains: functioning, fatigue/mood, fears/shame, and nutrition [12]. Scores range from 0 to 100, with higher scores indicating greater impairment. Responses reflect the patient's experiences over the preceding 28 days. A reduction in AE‐QoL total and domain scores indicate improved HRQoL. For the AE‐QoL total score, a 6‐point decrease was considered the MCID [13].

### 2.4 Statistical Methods

All analyses were conducted post hoc and were not pre‐specified in the original trial protocols or trial registration records. Baseline demographics and AE‐QoL scores were summarised using descriptive statistics (mean, standard deviation [SD], median, and interquartile range [IQR]). A mixed effects model for repeated measures (MMRM) assessed changes from baseline in AE‐QoL scores, comparing berotralstat with placebo through Week 24. The model included baseline AE‐QoL score, investigator‐confirmed HAE attack rate, treatment, visit, and visit‐by‐treatment interaction as fixed effects, with patient as a random effect. LSMD, 95% confidence intervals (CIs), and *p*‐values were reported; a two‐sided *p*‐value < 0.05 was considered statistically significant. No adjustment was made for multiple comparisons.

Long‐term changes in AE‐QoL total and domain scores were evaluated from baseline to Week 96 in the berotralstat cohort. In the absence of a placebo comparator after Week 24, the effect size of the change from baseline at each follow‐up visit was assessed per Cohen's *d* and calculated using a within‐subjects design per Lakens 2013 [14]. A Cohen's *d* value of 0.20–0.49 indicates a small effect, 0.50–0.79 a medium effect, and ≥ 0.80 a large effect. Median time to AE‐QoL deterioration and associated 95% CI were described using Kaplan–Meier curves.

The proportion of patients achieving MCID in AE‐QoL total score was calculated at Week 24 for the placebo cohort and at Weeks 24, 48, 72, and 96 for the berotralstat cohort. The difference in the proportion of patients achieving MCID in AE‐QoL total score between cohorts at Week 24 was assessed using logistic regression, adjusting for baseline AE‐QoL and HAE attack rate. As there is no established MCID threshold for AE‐QoL domain scores, meaningful change for domain scores per the distributional criterion method was defined as ≥ 0.5 SD in the direction of improvement, using the baseline SD derived from the pooled berotralstat and placebo cohorts [15]. The proportion of patients in the berotralstat cohort achieving clinically meaningful improvement for domain scores was assessed at Weeks 24, 48, 72, and 96. All analyses were conducted post hoc.

## 3 Results

### 3.1 Baseline Characteristics

A total of 47 patients treated with berotralstat (40 from APeX‐2; 7 from APeX‐J) and 45 treated with placebo (39 from APeX‐2; 6 from APeX‐J) were included in the analysis. The mean age was 39.6 years in the berotralstat cohort and 44.2 years in the placebo cohort. Most patients in both groups were female (61.7%; 68.9%) and White (80.9%; 82.2%) (Table 1).

**TABLE 1:** Baseline patient demographics and characteristics.

	Berotralstat 150 mg	Placebo
	(*N* = 47)	(*N* = 45)
Age at time of consent, years, mean (SD)	39.6 (13.3)	44.2 (14.1)
Age categories, *N* (%)		
12–17 years	2 (4.3%)	2 (4.4%)
18–64 years	44 (93.6%)	40 (88.9%)
≥ 65 years	1 (2.1%)	3 (6.7%)
Female sex, *N* (%)	29 (61.7%)	31 (68.9%)
Race, *N* (%)		
White	38 (80.9%)	37 (82.2%)
Asian	6 (12.8%)	6 (13.3%)
Other	3 (6.4%)	2 (4.4%)
Ethnicity, *N* (%)		
Hispanic or Latino	3 (6.4%)	1 (2.2%)
Not Hispanic or Latino	44 (93.6%)	43 (95.6%)
Unknown/Not Reported	0 (0.0%)	1 (2.2%)
Weight (kg), mean (SD)	83.0 (22.1)	83.3 (20.9)
BMI (kg/m^2^), mean (SD)	29.2 (7.1)	29.2 (6.7)
Weight based on BMI, *N* (%)		
Normal Weight	14 (29.8%)	14 (31.1%)
Overweight	16 (34.0%)	16 (35.6%)
Obese	17 (36.2%)	15 (33.3%)

Abbreviation: BMI, body mass index.

### 3.2 Comparison of AE‐QoL Scores Between Berotralstat and Placebo at Week 24

**FIGURE 1:**
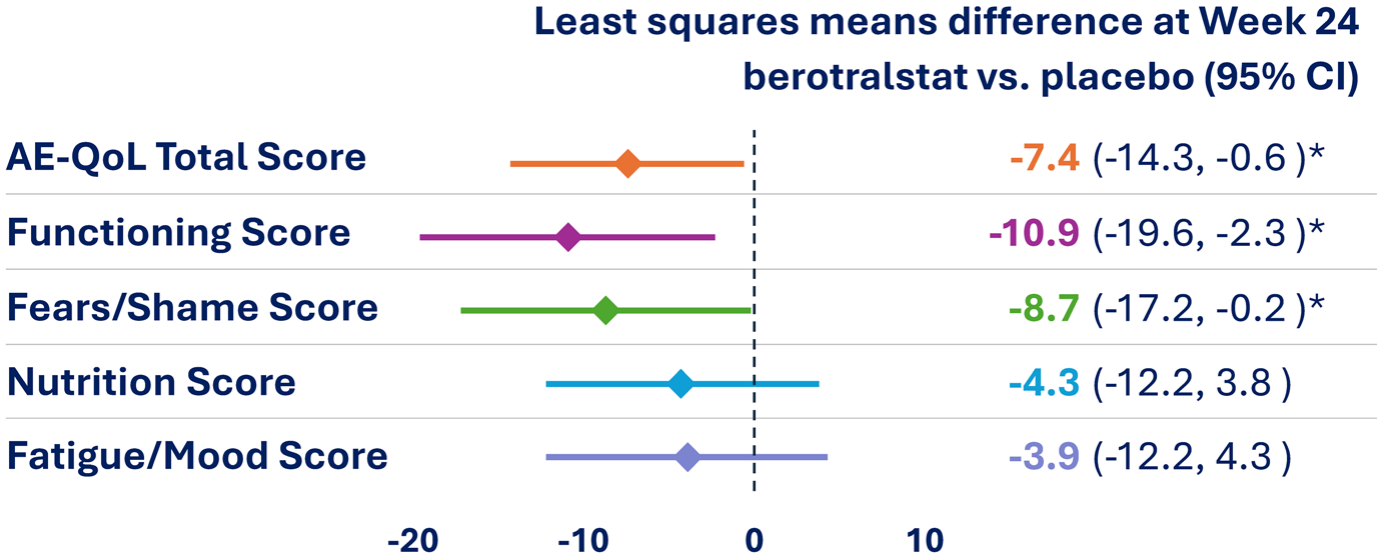
Comparison of AE‐QoL score between berotralstat and placebo at Week 24. AE‐QoL, Angioedema Quality of Life Questionnaire; CI, confidence interval; LS, least squares. *Indicates a *p*‐value < 0.05.

In both the berotralstat and placebo cohorts, mean (SD) changes from baseline to Week 24 in AE‐QoL total and domain scores were negative, indicating improved HRQoL post treatment (Table 2). By Week 24, patients receiving berotralstat demonstrated statistically significant greater improvements versus placebo in total score (LSMD: −7.4; 95% CI: −14.3, −0.6; *p* < 0.05). Patients receiving berotralstat also had significantly greater improvements in functioning domain (LSMD: −10.9; 95% CI: −19.6, −2.3; *p* = 0.013) and fears/shame domain (LSMD: −8.7; 95% CI: −17.2, −0.2; *p* = 0.046) scores compared with patients receiving placebo (Figure 1).

**TABLE 2:** Summary of AE‐QoL scores at baseline and Week 24.

	Berotralstat 150 mg				Placebo			
	Observed		Change from baseline		Observed		Change from baseline	
	*N*	Mean (SD)	*N*	Mean (SD)	*N*	Mean (SD)	*N*	Mean (SD)
AE‐QoL Total Score								
Baseline^a^	47	42.5 (18.0)	—	—	45	45.2 (19.6)	—	—
Week 24	45	27.1 (21.4)	45	−15.7 (20.8)	42	35.8 (15.6)	42	−10.3 (19.9)
Functioning Score								
Baseline^a^	47	46.4 (21.9)	—	—	45	43.6 (23.6)	—	—
Week 24	45	24.0 (24.1)	45	−22.3 (27.9)	42	33.7 (21.2)	42	−10.0 (25.7)
Fatigue/Mood Score								
Baseline^a^	47	36.0 (19.6)	—	—	45	42.9 (22.8)	—	—
Week 24	45	26.6 (23.0)	45	−10.1 (22.7)	42	34.3 (20.2)	42	−10.5 (24.1)
Fears/Shame Score								
Baseline^a^	47	49.3 (24.5)	—	—	45	52.9 (25.9)	—	—
Week 24	45	31.4 (27.4)	45	−18.2 (26.2)	42	41.6 (22.7)	42	−12.0 (23.7)
Nutrition Score								
Baseline^a^	47	30.9 (24.6)	—	—	45	31.1 (25.0)	—	—
Week 24	45	21.7 (25.1)	45	−9.4 (25.6)	42	25.9 (22.0)	42	−5.7 (23.8)

Abbreviations: AE‐QoL, Angioedema Quality of Life Questionnaire; SD, standard deviation.

^a^ Baseline was defined as the last non‐missing value occurring prior to initiation of study drug.

### 3.3 Long‐Term Impact of Berotralstat Prophylaxis

**FIGURE 2:**
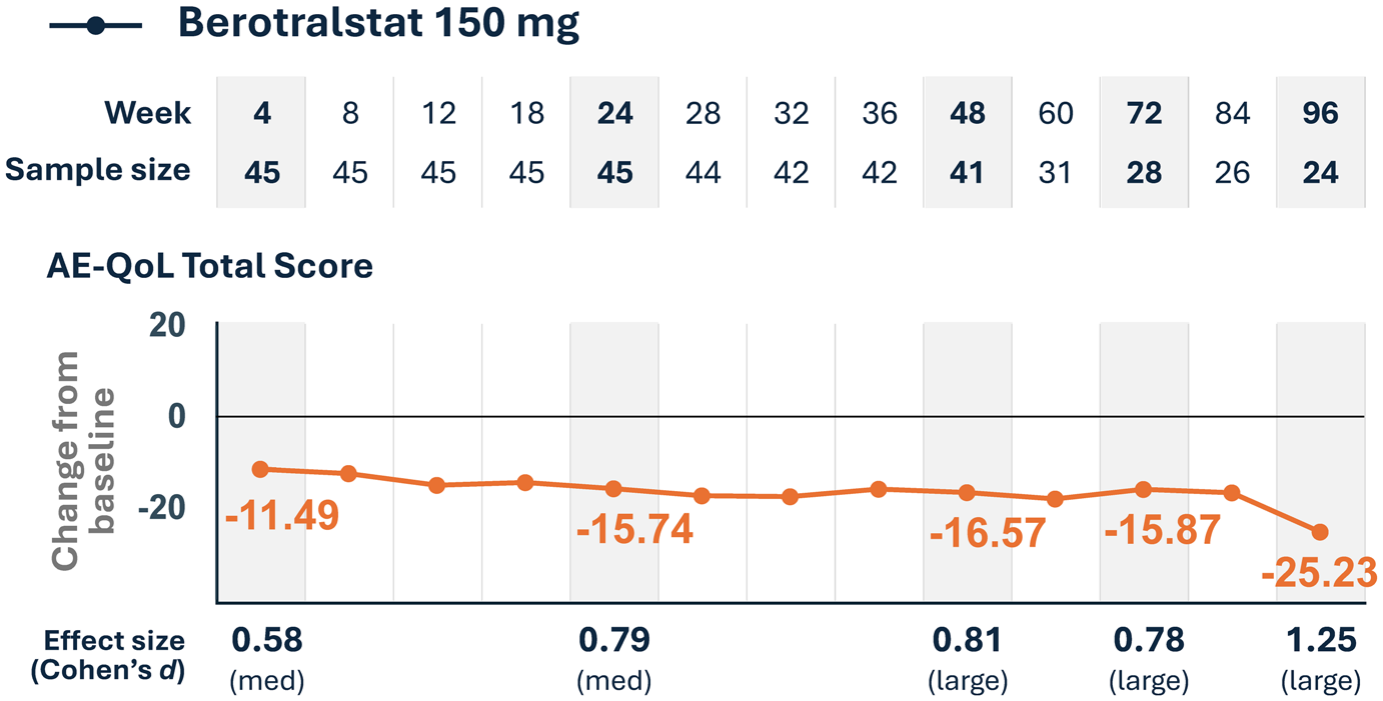
Change from baseline of AE‐QoL total score up to Week 96. Values represent mean change from baseline at Weeks 24, 48, 72, and 96. Negative values indicate improvement. Abbreviations: AE‐QoL: Angioedema Quality of Life Questionnaire; med: medium.

**FIGURE 3:**
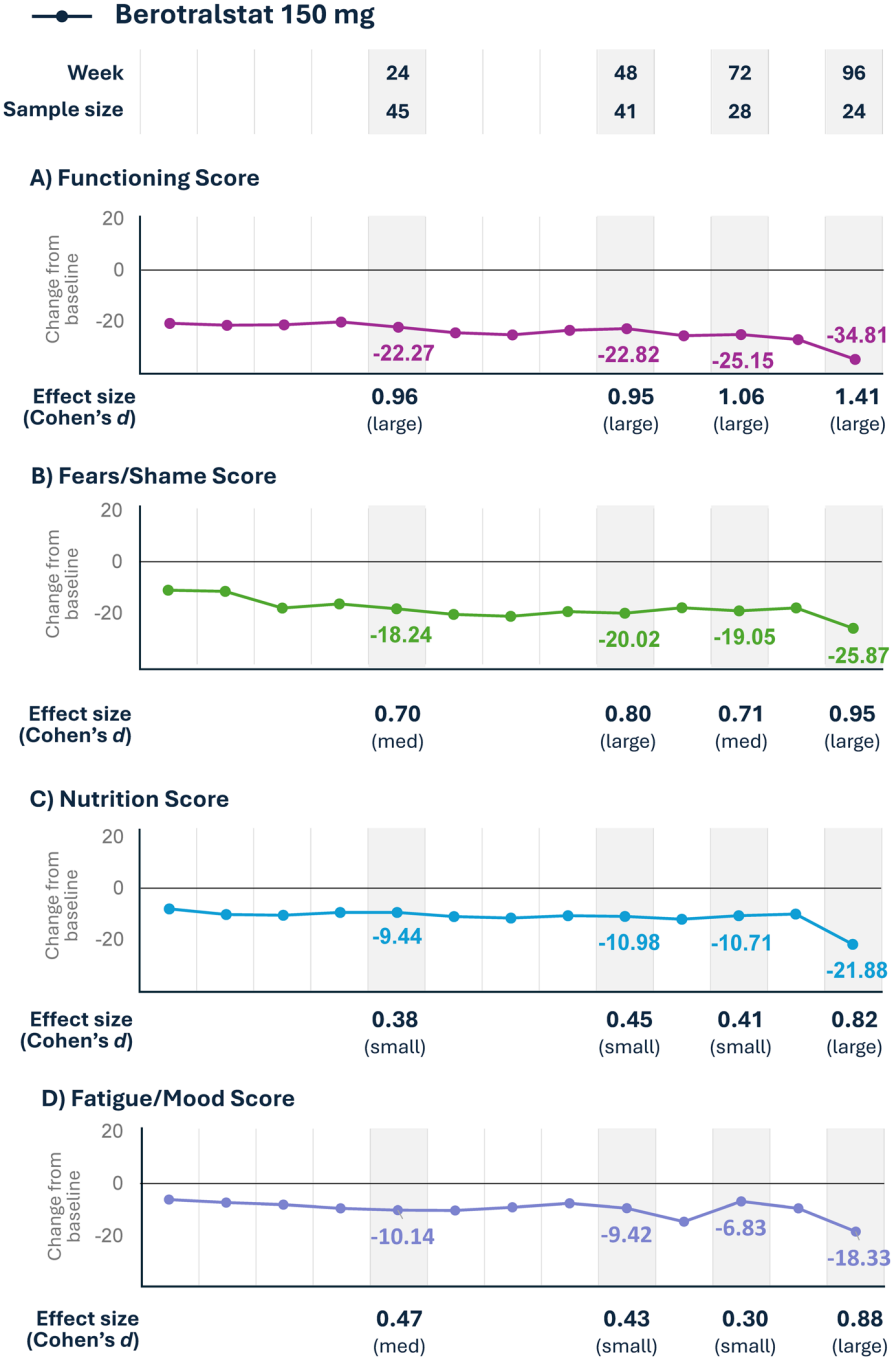
Change from baseline of AE‐QoL domain scores up to Week 96. (A) Functioning score; (B) Fears/Shame score; (C) Nutrition score; (D) Fatigue/Mood score. Values represent mean change from baseline at Weeks 24, 48, 72, and 96. Negative values indicate improvement. Abbreviations: AE‐QoL: Angioedema Quality of Life Questionnaire; med: medium.

Among patients receiving berotralstat, improvement in AE‐QoL total score observed at Week 24 was sustained through Week 96, with mean change at Week 96 of −25.2. The magnitude of improvement from baseline in AE‐QoL total scores was consistent with a medium effect size at Week 24, and with a large effect size at Week 48 and beyond based on Cohen's *d* (Figure 2). Consistent sustained improvements were observed across the functioning and fears/shame domain scores up to Week 96 (Figure 3).

### 3.4 Clinically Meaningful Improvement in AE‐QoL Scores

**FIGURE 4:**
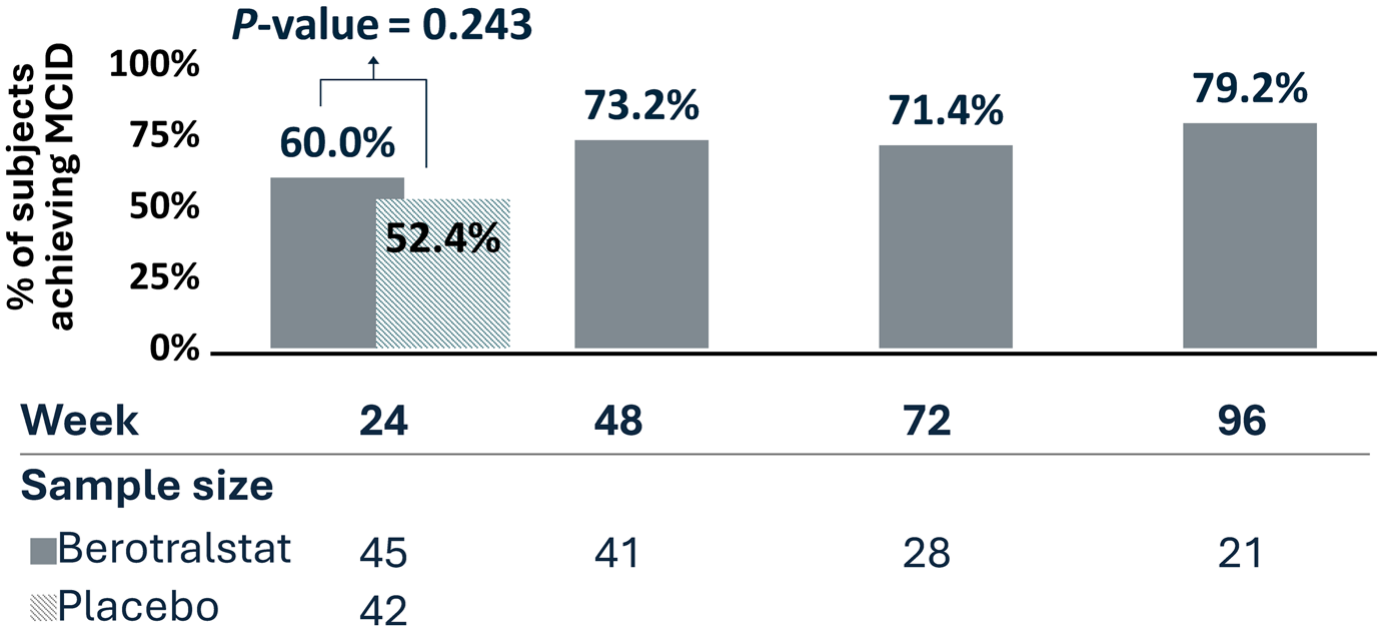
Improvement in AE‐QoL total score per MCID^1^. AE‐QoL, Angioedema Quality of Life Questionnaire; MCID, Minimal clinically important difference. [1] MCID for AE‐QoL was at least a 6‐point decrease from baseline AE‐QoL total score.

At Week 24, the proportion of patients achieving the MCID on the AE‐QoL total score was higher in berotralstat (60.0%) compared with placebo (52.4%), though the difference was not statistically significant. However, over the long term, the proportion of patients achieving MCID generally increased over time with berotralstat (Figure 4).

**FIGURE A1:**
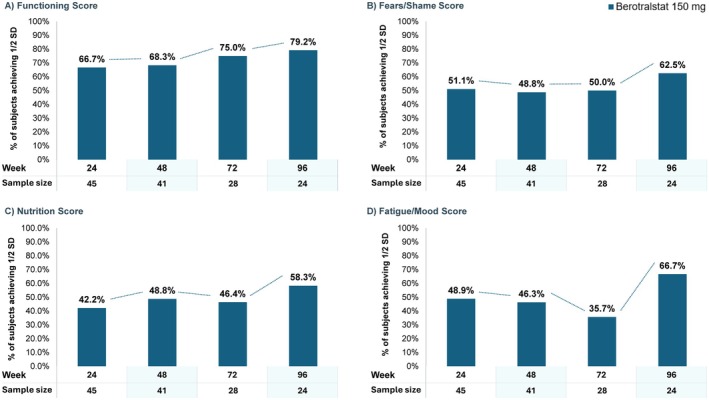
Improvement over time in AE‐QoL domain scores per distributional criterion method^1^ for the berotralstat cohort. AE‐QoL, Angioedema Quality of Life Questionnaire; SD, standard deviation. [1] Define as having change from baseline that achieves a decrease greater than half of the SD of the baseline value where the SD value was based on the baseline value of the pooled berotralstat 150 mg cohort for each AE‐QoL domain score.

The proportion of patients achieving meaningful improvements using the distributional criterion method in the functioning domain increased over time. For the fears/shame domain, the proportion reached 51.1% at Week 24, remained relatively stable through Week 72, and then increased through Week 96. For the nutrition domain and the fatigue/mood domains, the proportion of patients achieving improvements fluctuated over time but with overall increases in scores (Figure A1).

## 4 Discussion

Building on prior evidence from the APeX‐2 and APeX‐J clinical trials, this pooled analysis of individual patient data provides a robust evaluation of the effects of berotralstat 150 mg on HRQoL in patients with HAE. At Week 24, patients receiving berotralstat demonstrated significantly greater improvement versus placebo in AE‐QoL total (LSMD: −7.4; 95% CI: −14.3, −0.6; *p* < 0.05), functioning domain (LSMD: −10.9; 95% CI: −19.6, −2.3; *p* = 0.013), and fears/shame domain (LSMD: −8.7; 95% CI: −17.2, −0.2; *p* = 0.046) scores. Among patients receiving berotralstat, the benefits were sustained, with mean changes in AE‐QoL total score from baseline ranging from −15.7 to −25.2 across time points through Week 96, and effect sizes increasing from medium at Week 24 to large from Week 48 onwards. The improvements were clinically meaningful, with 60% of patients in the berotralstat group achieving MCID in AE‐QoL total score by Week 24 and the proportion of patients achieving MCID generally increasing over time. Sustained improvements were also observed in the functioning and the fears/shame domains based on distributional criterion. Taken together, these findings highlight the durable and multidimensional impact of prophylactic therapy with berotralstat on key aspects of patients' daily life.

Although data on the impact of HAE on HRQoL remain limited, the available evidence shows that the disease imposes a substantial physical, emotional, and social burden. Berotralstat improves quality of life by reducing attack frequency, alleviating anxiety linked to the unpredictability of the attacks, and supporting daily functioning.

In a US survey of 445 patients with HAE, frequent attacks were significantly associated with worse AE‐QoL scores [16]. Similarly, a Swedish study (*N* = 103) reported significantly poor perceived health among patients experiencing ≥ 30 attacks per year [17]. In APeX‐2 and APeX‐J, berotralstat significantly reduced attack frequency versus placebo, as early as the first month of treatment, with sustained efficacy through Week 24 [7, 11]. These reductions in disease activity likely contribute to the clinically meaningful HRQoL improvements observed in this analysis.

Emotional burden is also a major component of HAE, with high rates of anxiety and depression reported in several studies [18, 19, 20]. As the fears/shame domain of the AE‐QoL captures this burden [12], the improvements observed in our study indicate that berotralstat helps alleviate emotional distress by stabilising HAE attack patterns and lowering perceived risk of future episodes.

HAE impacts daily functioning even between attacks, affecting work productivity and participation in daily activities [16, 18, 19, 21]. In a European study, missed days from work or daily activities increased with attack frequency, ranging from 2.2 to 11.6 per month [21]. A survey of US‐based patients with HAE found that facial swelling episodes are associated with workplace absenteeism, reduced productivity, and social withdrawal [16]. The findings underscore the clinical value of prophylactic treatments like berotralstat, which may help preserve daily functioning by reducing attack frequency, as well as decreasing the negative impact of HAE between attacks.

Within the evolving landscape of long‐term prophylaxis for hereditary angioedema, berotralstat represents an important therapeutic option among available prophylactic approaches. As the only recommended orally administered first‐line prophylactic therapy for HAE, berotralstat eliminates the burden of parenteral administration, which can deter adherence owing to discomfort, preparation, and logistical barriers [22, 23, 24]. For appropriate patients, berotralstat offers a convenient alternative that may reduce treatment‐related burden and support adherence. In clinical trials, berotralstat improved patient satisfaction among individuals switching from injectable prophylaxis [8, 10, 25]. These practical advantages, alongside the sustained HRQoL improvements observed in this study, support the role of berotralstat as an effective and patient‐centered approach to optimising prophylaxis in HAE.

This study has several strengths. By pooling individual patient data from two phase 3 trials (APeX‐2 and APeX‐J), it enabled a robust evaluation of the short‐ and long‐term impact of berotralstat on HRQoL. The use of a validated, disease‐specific HRQoL instrument (AE‐QoL) ensured sensitivity to clinically relevant changes over time. Finally, analysis of domain‐level AE‐QoL data provided a nuanced understanding of how berotralstat affects distinct aspects of patients' everyday experiences, particularly emotional well‐being and daily functioning.

Limitations of this study include the short placebo‐controlled period (24 weeks) and the decreasing sample size over time owing to attrition, particularly after Week 48. Patients with inadequate disease control, adverse events, or persistently poor HRQoL may have been more likely to discontinue treatment; therefore, those remaining in the study at later time points may disproportionately represent individuals with better treatment response and improved quality of life. As a result, long‐term outcomes (Week 72 or later) may overestimate the durability and magnitude of treatment benefit, underscoring the need for further research to confirm the long‐term impact of berotralstat. This post hoc analysis included a few adolescents, which may affect result interpretation, as the AE‐QoL was originally developed and formally validated in adults (≥ 18 years). Nevertheless, the adoption of AE‐QoL in adolescent clinical trials and the recent content validation study support the relevance and applicability of AE‐QoL in adolescent patients with HAE [26, 27, 28]. Differences in population size and demographics between APeX‐2 and APeX‐J may have introduced heterogeneity. Lastly, as this was a post hoc exploratory analysis without adjustment for multiple comparisons, findings about statistical significance should be interpreted accordingly.

Notwithstanding the above limitations, the results of this analysis have important clinical implications. They provide relevant insights into the benefits of berotralstat in addressing the physical and emotional burden of HAE, supporting healthcare professionals in making informed treatment decisions that prioritize disease control as well as patient quality of life.

## 5 Conclusion

In this post hoc analysis, patients with HAE receiving berotralstat 150 mg experienced significantly greater improvements in AE‐QoL total, functioning domain, and fears/shame domain scores at Week 24, compared with those on placebo. The findings underscore the substantial impact of effective prophylaxis on both daily activities and psychological well‐being. Over the long term, patients receiving berotralstat 150 mg demonstrated sustained and progressively greater improvement, with a generally steady proportion of patients achieving clinically meaningful improvements. These HRQoL benefits were maintained through Week 96, reinforcing the long‐term value of berotralstat in HAE management.

Taken together, the findings support the use of berotralstat as a long‐term prophylactic therapy for HAE. Further validation in real‐world studies is needed to confirm these findings beyond the clinical trial setting.

## Author Contributions

T.C., S.K.‐A., D.T.J., A.W., L.L.‐G., Z.‐Y.Z., F.Y., S.N.‐P., P.G., and T.K. contributed to the conceptualization and design of the study, result interpretation, and critically reviewed and revised the manuscript. D.T. led the data analyses. H.H.K. contributed to result interpretation. All authors contributed to reviewing, editing, and approving the final manuscript.

## Funding

This study was funded by BioCryst Pharmaceuticals Inc. The study sponsor was involved in the study design, data collection, interpretation, and decision to submit the manuscript for peer review.

## Ethics Statement

This post hoc analysis was conducted using de‐identified data from a previously completed clinical trial. The original trials were approved by relevant regulatory authorities and institutional review boards/independent ethics committees and conducted in accordance with the Declaration of Helsinki and Good Clinical Practice guidelines. As this was a secondary analysis of existing data, no additional IRB approval was required for the current study.

## Conflicts of Interest

Teresa Caballero is or recently was a speaker, advisor, engaged in research or educational projects, received research grants and/or received funding to attend conferences or educational events from Astria, BioCryst, CSL Behring, Ionis, KalVista, Novartis, Otsuka, Pharvaris, and Takeda. She is a researcher on the Hospital La Paz Health Research Institute (IdiPAZ) Program to promote research activities. Sorena Kiani‐Alikhan is a chief and principal investigator for studies and in receipt of honorarium for consulting work and advisory boards organised by Shire/Takeda, CSL Behring, BioCryst Pharmaceuticals, Biotest, KalVista, Pharvaris, Astria, Ionis, X4 Pharmaceuticals, and Otsuka. Douglas T. Johnston, Sandra Nestler‐Parr, and Patrick Gillard are employees of and own stock/stock options in BioCryst Pharmaceuticals Inc. Lorena Lopez‐Gonzalez is a former employee of BioCryst Pharmaceuticals Inc. Dianne Tomita is a former employee of BioCryst Pharmaceuticals Inc. and holds stock in the company. Aolin Wang, Zheng‐Yi Zhou, Fan Yang, and Hannah H. Kim are employees of Analysis Group Inc., which received consultancy fees from BioCryst Pharmaceuticals Inc. for this work. Tamar Kinaciyan is on the advisory boards for BioCryst Pharmaceuticals, CSL Behring, KalVista Pharmaceuticals, and Takeda; is the clinical trial investigator for BioCryst Pharmaceuticals, KalVista Pharmaceuticals, Kiniksa Pharmaceuticals, and Takeda; received grants or honoraria from BioCryst Pharmaceuticals, CSL Behring, Novartis, and Takeda; and is on the speakers bureau for BioCryst Pharmaceuticals, CSL Behring, Novartis, and Takeda.

## Data Availability

The data that support the findings of this study will be made available upon reasonable request.
